# Forecasting Frequent Alcohol Use among Adolescents in HBSC Countries: A Bayesian Framework for Making Predictions

**DOI:** 10.3390/ijerph19052737

**Published:** 2022-02-26

**Authors:** Lorena Charrier, Michela Bersia, Alessio Vieno, Rosanna Irene Comoretto, Mindaugas Štelemėkas, Paola Nardone, Tibor Baška, Paola Dalmasso, Paola Berchialla

**Affiliations:** 1 Department of Public Health and Pediatrics, University of Torino, 10126 Torino, Italy; lorena.charrier@unito.it (L.C.); michela.bersia@edu.unito.it (M.B.); paola.dalmasso@unito.it (P.D.); 2 Post Graduate School of Medical Statistics, University of Torino, 10126 Torino, Italy; 3 Department of Developmental and Social Psychology, University of Padova, 35131 Padova, Italy; alessio.vieno@unipd.it; 4 Health Research Institute, Faculty of Public Health, Lithuanian University of Health Sciences, 47181 Kaunas, Lithuania; mindaugas.stelemekas@lsmuni.lt; 5 Department of Preventive Medicine, Faculty of Public Health, Lithuanian University of Health Sciences, 47181 Kaunas, Lithuania; 6 National Centre for Disease Prevention and Health Promotion, Italian National Institute of Health, 00161 Rome, Italy; paola.nardone@iss.it; 7 Department of Public Health, Jessenius Faculty of Medicine in Martin, Comenius University in Bratislava, 036 01 Martin, Slovakia; tibor.baska@uniba.sk; 8 Department of Clinical and Biological Sciences, University of Torino, 10043 Orbassano, Italy; paola.berchialla@unito.it

**Keywords:** adolescents, alcohol, drinking, risk behaviour, trend, forecast, Bayesian longitudinal model

## Abstract

(1) Aim: To summarize alcohol trends in the last 30 years (1985/6–2017/8) among 15-year-olds in Health Behaviour in School-aged Children (HBSC) countries (overall sample size: 413,399 adolescents; 51.55% girls) and to forecast the potential evolution in the upcoming 2021/22 HBSC survey. (2) Methods: Using 1986–2018 prevalence data on weekly alcohol consumption among 15-year-olds related to 40 HBSC countries/regions, a Bayesian semi-parametric hierarchical model was adopted to estimate trends making a clusterization of the countries, and to give estimates for the 2022 HBSC survey. (3) Results: An overall declining trend in alcohol consumption was observed over time in almost all the countries. However, compared to 2014, some countries showed a new increase in 2018 and 2021/22 estimates forecast a slight increase in the majority of countries, pointing out a potential bounce after a decreasing period in frequent drinking habits. (4) Conclusions: The clusterization suggested a homogenization of consumption habits among HBSC countries. The comparison between 2022 observed and expected data could be helpful to investigate the effect of risk behaviour determinants, including the pandemic impact, occurring between the last two waves of the survey.

## 1. Introduction

Excessive alcohol use has been identified as one of the major preventable risk factors for population health globally having a significant impact on many health-related indicators, as pointed out by the United Nations (UN) Sustainable Development Goals (SDGs) target for “Strengthen the prevention and treatment of substance abuse, including narcotic drug abuse and harmful use of alcohol” [1]. The WHO’s Non-Communicable Disease (NCD) Global Monitoring Framework sets a voluntary target to reduce the harmful use of alcohol as one of the NCD reduction aims till year 2025 [2].

The high public health burden of alcohol consumption is particularly critical in the young age group (15–19 years old), where alcohol attributable mortality accounts for around one-fifth of all deaths [3]. In addition to fatal consequences, adolescent alcohol use is often associated with other risky behaviours, such as other substance use and unprotected sex; the literature confirms the co-occurrence of multiple health risk behaviours and the idea that they tend to cluster together [4,5]. Negative psychological, social and physical health effects (school failure, violence, injuries, suicide attempts, sexually transmitted diseases and unintended pregnancy, among others) have also been observed. Moreover, the earlier the drinking initiation, the more severe the consequences, with an increased risk of alcohol abuse and a greater likelihood of substance use disorders in later life [6].

Adolescent alcohol use in Europe has shown a declining tendency in recent decades [7,8] even if it has not followed homogeneous patterns among countries. This trend initially was not noticeable in Central and Eastern Europe and consumption even increased in some countries between 1998 and 2006 [9,10]. However, since 2006, the decline became apparent in other parts of the continent [11,12,13]. The decline was more pronounced in boys leading to a reduction in their traditional predominance above girls. Because of the mentioned changes over decades, heterogeneity across Europe has become less noticeable, and countries now show a more uniform picture. Such globalizing development of alcohol use among adolescents [14,15] can be caused by the gradual predominance of global factors over local ones. In fact, similar trends have been observed outside Europe, in Australia, New Zealand, Canada, Japan and USA [13].

Despite the recent declines, adolescent frequent alcohol consumption remains a major public health concern. In fact, because of its availability, alcohol is still one of the most used drugs among young people [6]. Monitoring trends is thereby crucial to early detect changes over time and promptly implement tailored health promotion interventions. Moreover, the above-indicated global targets are closely associated with specific indicators such as alcohol per capita consumption or heavy episodic drinking among adults and adolescents. The Health Behaviour in School-aged Children (HBSC) study serves as an important source for several indicators to measure alcohol-related challenges relevant to both country-specific and regional assessment of health-related behaviours among adolescents (www.hbsc.org, accessed on 14 January 2022).

This study aims to summarize the HBSC alcohol-related data of the last three decades and forecast the trends in frequent alcohol consumption among 15-year-olds in the upcoming HBSC survey sample as a contribution to the monitoring process of this public health issue.

## 2. Materials and Methods

### 2.1. Participants

The HBSC surveillance is a World Health Organization (WHO) collaborative cross-national study that monitors adolescent health and well-being every 4 years in more than 50 European and North American countries and regions. It is a school-based survey that uses a self-report questionnaire administered to whole school classes. Data from each country are collected according to the HBSC internationally approved protocol for each survey round to ensure consistency in survey instruments (such as a list of mandatory questions), data collection and processing procedures [16,17]. Samples are designed to be nationally representative of pupils aged 11, 13 and 15 years. Data for this study are based on 15-year-old adolescents, the age group with higher alcohol consumption prevalence [6].

For our analyses, we used HBSC data collected by the 40 countries that took part in at least three out of nine survey waves from 1985/6 and that had comparable data on alcohol consumption collected through the same question, mandatory until 2013/14, for at least one of the last two surveys (2013/14 and 2017/18). The countries/regions that met the inclusion criteria (see also Table A1 in Appendix A) were: Armenia (AM), Austria (AT), Flemish Belgium (BE-VLG), French Belgium (BE-WAL), Bulgaria (BG), Canada (CA), Switzerland (CH), Czechia (CZ), Germany (DE), Denmark (DK), Estonia (EE), Spain (ES), Finland (FI), France (FR), England (GB-ENG), Scotland (GB-SCT), Wales (GB-WLS), Greenland (GL), Greece (GR), Croatia (HR), Hungary (HU), Ireland (IE), Israel (IL), Iceland (IS), Italy (IT), Lithuania (LT), Luxembourg (LU), Latvia (LV), former Yugoslav Republic of Macedonia (MK), Malta (MT), Netherland (NL), Norway (NO), Poland (PL), Portugal (PT), Romania (RO), Russian Federation (RU), Sweden (SE), Slovenia (SI), Slovakia (SK), and Ukraine (UA).

Analyses were stratified by gender due to the differences in both prevalence and trend of frequent alcohol consumption between boys and girls.

### 2.2. Measures

#### Weekly/Daily Alcohol Use

Weekly/daily alcohol use was evaluated with the question ‘At present, how often do you drink anything alcoholic? Try to include even those times when you only drink a small amount’. The items were beer, wine, spirits, alcopops, aperitifs, cider, cocktails and other when asked. For each item, response options were ‘1 = every day’, ‘2 = every week’, ‘3 = every month’, ‘4 = rarely’ and ‘5 = never’. An overall alcohol use index was created considering the highest frequency of any alcoholic beverage consumed. The variable was dichotomized into ‘at least weekly alcohol use’ (response options 1 and 2) and ‘less than weekly’ (response options 3, 4 and 5).

The variable construction took into account the changes over time in the items included in the question mentioned above, due to changes in the consumption of specific alcoholic drinks: wine, beer and spirits have been present since the beginning of HBSC survey, cider was included only until the 1993/94 wave, while alcopops and ‘other’ have been added since the 2005/06 survey.

In 2017/18, due to the modification of some mandatory questions, data on frequent alcohol consumption comparable to previous waves were available in only 23 of the 40 countries/regions included in this study: Armenia (AM), Austria (AT), Flemish Belgium (BE-VLG), French Belgium (BE-WAL), Bulgaria (BG), Switzerland (CH), Czechia (CZ), Denmark (DK), Estonia (EE), Scotland (GB-SCT), Wales (GB-WLS), Croatia (HR), Ireland (IE), Israel (IL), Italy (IT), Lithuania (LT), Latvia (LV), Malta (MT), Norway (NO), Russian Federation (RU), Sweden (SE), Slovenia (SI), and Slovakia (SK).

### 2.3. Statistical Analysis

A Bayesian semi-parametric hierarchical model was adopted to estimate the alcohol weekly consumption trend in each of the 40 included countries. Following the model introduced by Paddock et al. 2013 [18] for the analysis of longitudinal data,

(1) $$y_{ij}=\alpha +f\left(t_{ij}\right)+g_{i}(t_{ij})+\epsilon_{ij}$$

where $y_{ij}$ represents the alcohol weekly consumption observed for country i at wave $j=\left(1,\ldots ,m_{i}\right)$ and $m_{i}$ is the number of waves the i-th country participated in and varies country-by-country. The term $f(t_{ij})$ is a polynomial function of time to allow for non-linear trends and it takes the form $\left(t,\;t_{ij},\;t_{ij}{}^{2},\ldots ,\;t_{ij}{}^{n}\right)\beta$, with β being the vector of the fixed effect coefficients. Analogously, $g_{i}(t_{ij})$ takes the form $\left(t,\;t_{ij},\;t_{ij}{}^{2},\ldots ,\;t_{ij}{}^{n}\right)b_{i}$, with $b_{i}$ being the set of the random effects (country-level) coefficients.

To be as flexible as possible in modelling the trend, we adopted the Dirichlet Process (DP) framework introduced by Escobar and West [19] that generalizes the prior construction on random effects $b_{i}$ to non-parametric formulation, allowing estimation of a different form than a specified parametric distribution, typically Gaussian distribution.

Thus, the random effects are assumed to be generated from an unknown distribution *F* and are assumed to be conditionally independent, given *F*

(2) $$\;b_{1},\ldots b_{n}∼F,\;\mathrm{with}\;F|c,\;F_{o}∼DP\left(c,\;F_{0}\right)$$

$F_{0}$ represents the ‘best guess’ about the form of F prior to observing data and the precision parameter, *c* > 0, expresses the degree of confidence that $F_{0}$ is the correct generating distribution for random effects, i.e., the higher the value of c, the more F is expected to conform to $F_{0}$. $F_{0}$ was chosen as normal distribution $N\left(0,\;\tau^{-1}\right)$. Flat priors for fixed effects β and the global intercept α were considered. Independent Gamma(0.1, 0.1) are defined for the precision parameters τ.

The DP has the property to have a positive probability for ties to occur among the values of the random effects $b_{i}$. The set of unique values among the random effects are called clusters, and we exploited such properties of DP to borrow strength in trend estimation from countries who share the same random effects. Specifically, countries with similar trends will have higher probabilities of co-clustering.

We impose c∼Gamma (a, 1), which encodes the prior expected number of clusters (the higher the values for shape parameter a the higher the number of clusters).

To obtain the posterior estimates of the model parameters, we ran 100,000 MCMC iterations, with the first 30,000 as burn-in.

All the analyses were performed using R version 4.1.2 [20].

## 3. Results

The overall sample size was 413,399 adolescents from the 40 countries that met the inclusion criteria; the mean age was 15.5 years, and 51.55% were girls.

### 3.1. Prevalences of Weekly/Daily Alcohol Consumption from 1986 to 2018

Table 1 and Table 2 show the prevalence of weekly/daily alcohol consumption for each country and wave for boys and girls, respectively.

The data show an overall declining trend in frequent alcohol consumption over the last three decades, in almost all countries, both in boys and girls. For boys, the estimated trends showed an increase in alcohol consumption in the decade 1990–2000 followed by a decrease. A notable exception is Bulgaria (BG), for which, however, only the data of three survey waves (2000, 2014 and 2018) were available. The same pattern is observed among girls. However, Canada (CA) is a notable exception, as it can be observed that the estimated trend captures the decreasing trend starting from 2000, but it misses a decreasing trend also in the decade 1990–2000. Moreover, after a nadir in drinking behaviour in the 2013/14 HBSC wave, several countries/regions showed a new increase in 2017/18: Armenia (AM), Austria (AT), Flemish Belgium (BE-VLG), Bulgaria (BG), Denmark (DK), Wales (GB-WLS), Ireland (IE), Norway (NO), Sweden (SE), and Slovakia (SK) among boys and Austria (AT), Bulgaria (BG), Denmark (DK), Scotland (GB-SCT), Wales (GB-WLS), Croatia (HR), Ireland (IE), Lithuania (LT), Latvia (LV), and Sweden (SE) among girls. Countries in which the increase was more dramatic were Austria (AT), with +1.0 and +7.2%, among boys and girls, respectively, Bulgaria (BG), +17.3 and +17.4%, and Denmark (DK), with +4.9 and +5.3%.

### 3.2. 2021/22. Prediction of Drinking Behavior

**Figure 1:**
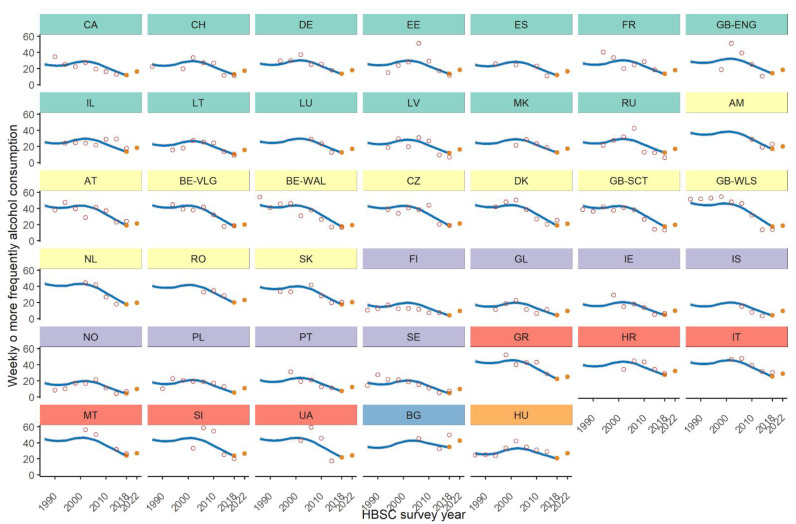
Trends (empty circles) and estimated (full circles for 2017/18 and 2021/22 waves) prevalences, (%), by country. Countries are grouped according to clusterization (different colors). Boys.

**Figure 2:**
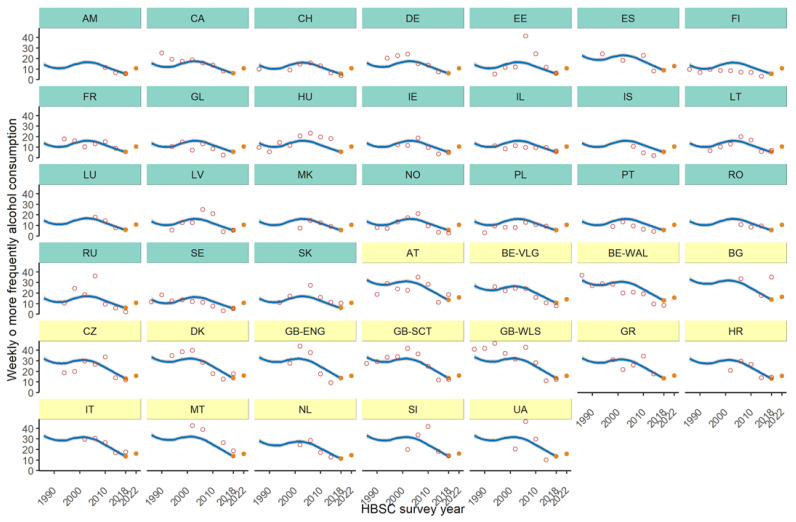
Trends (empty circles) and estimated (full circles for 2017/18 and 2021/22 waves) prevalences, (%), by country. Countries are grouped according to clusterization (different colors). Girls.

Figure 1 and Figure 2 depict, for boys and girls respectively, the trends over time of weekly/daily alcohol consumption. Clusterization of trends points out differences in the observed decreasing alcohol consumption among European countries. For boys, six clusters have been detected according to the presence of ties among random effects, which occur for countries with similar trends over time, and are displayed through different colors of the panels in figures. Only Bulgaria (BG) and Hungary (HU) do not clusterize with any other country. For girls, only two clusters made up of 16 and 24 countries have been detected, meaning more similar trends among countries.

Table 3 shows for each country the forecast of weekly/daily alcohol consumption in the next survey wave (2021/22) stratified by gender. The forecast is also displayed in Figure 1 and Figure 2 (last full orange circle in each country trend).

The comparison between the predicted 2021/22 and the observed 2017/18 alcohol consumption prevalence suggests that we will expect a slight increase in the majority of the countries/regions. However, Austria (AT), Bulgaria (BG), Denmark (DK) and Italy (IT) could show a reduction in drinking behaviour in both gender groups, while in Armenia (AM) it could happen only among boys.

### 3.3. Model Assessment. Comparison between 2017/18 Estimates and Observed Prevalences

We tested the model prediction on the 23 countries for which alcohol consumption data were available in the 2017/18 survey. Table 4 and Table 5 report for boys and girls, respectively, partial results from the clusterization of countries/regions and the absolute error (AE) of the model in estimating the 2017/18 weekly/daily alcohol consumption by country. The AE of the model was computed as the difference between the estimated and observed alcohol consumption for the 23 countries that still used the question “At present, how often do you drink anything alcoholic? Try to include even those times when you only drink a small amount” in the 2017/18 HBSC wave. Mean, median, minimum and maximum errors over countries are also reported.

The model provided for 2017/18 forecasting an overall median error of 2.82 for boys and 1.44 for girls. The biggest error of the model (14.89 for boys and 21.26 for girls) turned out to be that for Bulgaria (BG); this country met the inclusion criteria of the study but provided data from only three non-consecutive waves (2006, 2014 and 2018) for trends and subsequent estimates that could be an explanation of the huge AE of the model.

## 4. Discussion

The present study provided an approach to model longitudinal data, to estimate potentially nonlinear trends and forecast frequent alcohol consumption prevalence given knowledge from the past. We chose a Bayesian framework analysis due to its flexibility in relaxing assumptions on data distribution and intrinsically tying countries with similar trends. We used a data-driven dynamic clustering, which avoided us making an a priori guess on geographical patterns associated with alcohol consumption behaviours and, at the same time, borrowed strength from similar trend patterns in making estimations. That means that we nested the choice of the number of clusters inside the model and the grouping of the countries based on trend similarity. As can be further observed, the estimates of those countries with few survey waves’ data were possible due to the probability of co-clustering induced by the chosen modelling approach.

### 4.1. Clusters and Trends of Frequent Alcohol Consumption

Our results appear to confirm the findings of previous research about alcohol consumption patterns within Europe. Our clusterization keeps together some historically grouped countries/regions, such as Scandinavian countries, Baltic Republics, the Czech Republic and Slovakia, and Belgian regions. However, it is difficult to find robust geographical patterns, as the data show that not all countries within the same region share similar trends. This confirms that previous classifications of drinking habits based on regional cultures (i.e., ‘wet’ versus ‘dry’ cultures) and historical traditions (i.e., Italy labelled a “wine country” or Germany a “beer country”) are disappearing [21]. A homogenization of consumption rates and beverage preferences is increasingly evident: in recent decades, wine consumption has decreased in the traditionally ‘wet’ Mediterranean countries while overall alcohol consumption has increased in the northern European countries [14,22,23]. Moreover, it suggests that to explore adolescent drinking behaviour, it is necessary to take into account the role played by other factors, such as changing in social norms and ways in which young people socialize, inequalities (socioeconomic status, ethnic background), public health initiatives and policies enforced in each country (i.e., increasing taxes, age restrictions for purchasing alcohol, advertising regulations and availability restrictions) and also the strength of their implementation [24,25,26]. Furthermore, a recent systematic review explored the reasons for the decline of adolescent drinking and identified in the shifts in parental practices (parent–child relationship quality, parental support and involvement) the most robust evidence [27]. Finally, the indirect role of the global financial crisis of 2007–2009 should also be taken into account, and so should the COVID-19 pandemic when discussing data from the next survey.

### 4.2. Comparison between 2017/18 Estimates and Observed Prevalences

The Bayesian model we implemented has an acceptable error in forecasting alcohol consumption prevalence, as confirmed by the AE computed as the difference between the observed and the estimated prevalence in 2017/18 for the 23 countries that collected data through the same question used in the previous waves of the survey. Except for Bulgaria, with an AE of 14.89 and 21.26 for the prediction of alcohol consumption in boys and girls, respectively, only in other three countries (Russian Federation, Armenia and Denmark) is the AE greater than 5.1% for boys’ weekly consumption prediction

### 4.3. 2021/22. Forecasting of Frequent Alcohol Use

The forecasting of weekly/daily alcohol consumption in the next wave 2021/22 shows a slight increase in most countries for both boys and girls, pointing out a potential bounce after a period of decreasing in frequent drinking habits.

The comparison between the future observed prevalence with the expected one will offer the opportunity to further investigate the potential effect of determinants occurring in the period between the two most recent waves of the HBSC survey (2018–2022). This could be even more relevant for the assessment of the pandemic effect on risk behaviours. Studies dealing with this issue showed decreased alcohol drinking among adolescents during lockdowns [28]. However, the findings of an Italian study indicate that alcohol-related problems were even accentuated following reopening when compared with the same period in the previous year [29].

In this frame, the 2021/22 forecasts included in this study, and based on data from a thirty-year trend, could help explain any discrepancies with the prevalence of alcohol consumption observed after the pandemic.

### 4.4. Strengths and Limitations

Among the strengths of the study, the large sample and number of countries involved and the use of validated questionnaires that allow for cross-national comparisons can be accounted for. The study also has some limitations. First, the assessment of alcohol consumption is based on self-reported measures, so that alcohol consumption could be under-reported, especially among adolescents who are heavy drinkers. Second, the lack of data for some countries in specific years made it difficult to identify a robust temporal trend and to forecast the 2021/22 alcohol consumption prevalence. Moreover, the changes to the mandatory questions of the HBSC questionnaire meant that for the 2017/18 survey, only just over half of the countries had data comparable to the previous waves of the surveillance.

## 5. Conclusions

The impact of the COVID-19 pandemic and related lockdowns on adolescents’ risk conducts remains an open question. We can expect that increased use of online communication with limited possibilities of personal meetings and spending spare time outside influenced drinking behaviours and accented global determinants.

From a public health perspective, monitoring (and, hopefully, forecasting) trends in adolescent alcohol use and comparing predicted and observed data can help us to make hypotheses about the effectiveness of policies and health promotion interventions, and about the reasons for any discrepancies in alcohol consumption from the expected prevalence on the basis of a thirty-year trend.

Despite almost all countries having experienced a decline in frequent alcohol consumption in the last three decades, this decrease remains largely unexplained and adolescent alcohol use a major public health concern due to both short and long-term adverse outcomes it is associated with. Unlike other substances, such as tobacco, which in recent years has shown a reverse gender gap [30,31], habitual alcohol consumption remains more prevalent among boys than girls of the same age. Nevertheless, the data confirm a narrowing of the gender gap in many countries and regions.

Finally, further research is needed to better understand the factors associated with adolescent drinking and its changes over time.

## Figures and Tables

**Table 1:** Prevalence (%) of weekly/daily alcohol consumption by country and wave from 1985/86 to 2017/18 among boys.

Country ^1^	1985/86	1989/90	1993/94	1997/98	2001/02	2005/06	2009/10	2013/14	2017/18
AM							28.73	18.97	22.89
AT		37.83	47.3	39.57	28.82	41.25	37.3	22.85	23.86
BE-VLG			44.82	38.93	37.87	41.67	31.63	17.60	18.46
BE-WAL	54.01	40.95	45.87	45.95	30.91	37.85	26.4	17.02	16.17
BG						45.42		32.43	49.70
CA		34.49	24.98	22.37	27.2	19.12	16.14	12.78	
CH	22.24			19.64	33.42	26.98	26.51	11.36	11.17
CZ			38.73	33.74	40.58	38.7	44.01	20.38	18.93
DE			29.45	29.78	37.05	25.1	25.24	17.93	
DK			41.82	48.13	50.39	38.61	26.75	20.39	25.27
EE			14.71	23.48	28.11	51.01	29.04	16.95	11.34
ES			25.78		24.23		22.97	10.79	
FI	10.59	12.28	17.13	12.43	12.68	11.54	7.06	7.68	
FR			40.1	33.58	19.95	24.44	28.25	18.25	
GB-ENG				18.58	51.03	39.14	24.79	10.47	
GB-SCT	38.43	36.28	41.94	37.45	40.91	38.26	26.27	14.14	13.22
GB-WLS	51.57	51.92	52.94	54.48	47.98	45.9	31.48	13.45	13.95
GL			11.38	18.89	22.68	11.23	6.47	11.26	
GR				52.09	39.94	42.74	43.1	28.59	
HR					34.31	44.63	43.27	34.26	29.22
HU	24.48	25.06	23.93	33.23	42.31	34.68	31.01	29.06	
IE				29.26	14.97	17.96	13.6	5.11	6.52
IL			24.23	24.53	23.96	21.61	28.74	29.22	17.70
IS						15.08	7.87	3.36	
IT					46.83	47.68	39.33	31.55	30.31
LT			15.67	18.12	27.63	25.47	24.41	13.65	9.01
LU						29.23	23.44	12.58	
LV			18.64	29.41	19.78	31.03	26.68	9.46	6.77
MK					21.56	28.47	23.35	18.78	
MT					55.97	50		31.85	26.13
NL					44.53	42.29	27.03	18.01	
NO		8.47	9.95	17.05	16.71	21.41	10.99	4.06	6.85
PL		10.13	22.51	20.68	19.19	18.63	17.31	12.77	
PT				31.02	19.3	20.85	12.4	11.27	
RO						33.07	34.58	28.57	
RU			21.39	27.61	31.83	42.4	12.85	12.16	6.03
SE	14.34	27.55	21.76	21.29	18.73	15.35	10.63	5.08	7.53
SI					33.02	58.58	54.43	25.14	20.11
SK			33.78	33.33		41.97	28.19	19.73	20.35
UA					43	59.24	45.44	17.45	

^1^ AM (Armenia), AT (Austria), BE-VLG (Flemish Belgium), BE-WAL (French Belgium), BG (Bulgaria), CA (Canada), CH (Switzerland), CZ (Czechia), DE (Germany), DK (Denmark), EE (Estonia), ES (Spain), FI (Finland), FR (France), GB-ENG (England), GB-SCT (Scotland), GB-WLS (Wales), GL (Greenland), GR (Greece), HR (Croatia), HU (Hungary), IE (Ireland), IL (Israel), IS (Iceland), IT (Italy), LT (Lithuania), LU (Luxembourg), LV (Latvia), MK (former Yugoslav Republic of Macedonia.), MT (Malta), NL (Netherland), NO (Norway), PL (Poland), PT (Portugal), RO (Romania), RU (Russian Federation), SE (Sweden), SI (Slovenia), SK (Slovakia), UA (Ukraine).

**Table 2:** Prevalence (%) of weekly/daily alcohol consumption by country and wave from 1985/86 to 2017/18 among girls.

Country ^1^	1985/86	1989/90	1993/94	1997/98	2001/02	2005/06	2009/10	2013/14	2017/18
AM							11.61	6.68	5.48
AT		18.64	29.09	23.96	22.59	34.94	28.29	11.25	18.42
BE-VLG			25.86	22.31	24.44	24.12	15.79	10.74	7.94
BE-WAL	36.73	26.93	28.77	28.24	20.11	20.78	19.1	9.58	8.45
BG						33.37		17.65	35.09
CA		25.16	19.39	17.29	18.84	15.62	13.77	8.20	
CH	9.86			9.18	14.65	15.93	13.08	6.32	3.81
CZ			18.61	19.93	29.61	26.59	33.51	13.89	11.86
DE			20.35	22.72	24.27	15.21	13.83	7.42	
DK			35.01	38.56	39.91	28.3	17.72	12.52	17.81
EE			5.18	11.48	12.04	41.16	24.56	11.89	6.31
ES			24.52		18.36		22.78	8.21	
FI	9.76	6.95	9.58	8.56	8.45	7.17	6.81	3.33	
FR			17.82	16.01	10.47	13.31	15.38	8.95	
GB-ENG				27.75	43.72	37.82	17.6	9.20	
GB-SCT	27.34	29.17	33.43	33.81	41.49	36.54	24.75	11.90	12.32
GB-WLS	40.95	41.59	46.17	36.9	31.45	42.61	28.12	10.97	12.42
GL			10.61	15.1	7.14	13.27	8.47	2.58	
GR				31	21.64	25.79	34.47	17.61	
HR					20.79	29.75	26.68	13.86	14.31
HU	9.73	5.62	14.57	11.63	20.75	23.25	19.76	18.23	
IE				12.37	11.78	18.78	9.7	3.51	5.03
IL			11.42	8.56	11.49	9.77	9.63	9.76	6.60
IS						10.93	4.62	2.09	
IT					29.48	30.58	26.36	17.02	17.61
LT			6.76	10.5	13.01	20.04	16.81	6.01	7.12
LU						17.75	14.51	7.78	
LV			5.56	12.57	12.62	25.07	21.08	4.02	5.43
MK					7.28	14.76	12.64	9.09	
MT					42.51	39.02		26.48	18.70
NL					24.47	28.51	17.21	12.89	
NO		8.03	7.08	13.46	17.19	21.19	9.57	3.56	2.74
PL		2.95	9.56	8.09	7.83	12.87	10.71	9.27	
PT				8.97	13.45	9.57	6.42	4.24	
RO						10.7	8.37	9.38	
RU			10.51	24.51	18.43	36.12	9.29	5.64	2.07
SE	11.61	18.16	12.4	13.53	11.89	11.13	7.58	3.08	5.15
SI					19.92	33.61	41.78	18.27	14.17
SK			10.83	17.13		27.29	15.94	11.19	10.39
UA					20.49	46.47	29.93	10.16	

^1^ AM (Armenia), AT (Austria), BE-VLG (Flemish Belgium), BE-WAL (French Belgium), BG (Bulgaria), CA (Canada), CH (Switzerland), CZ (Czechia), DE (Germany), DK (Denmark), EE (Estonia), ES (Spain), FI (Finland), FR (France), GB-ENG (England), GB-SCT (Scotland), GB-WLS (Wales), GL (Greenland), GR (Greece), HR (Croatia), HU (Hungary), IE (Ireland), IL (Israel), IS (Iceland), IT (Italy), LT (Lithuania), LU (Luxembourg), LV (Latvia), MK (former Yugoslav Republic of Macedonia.), MT (Malta), NL (Netherland), NO (Norway), PL (Poland), PT (Portugal), RO (Romania), RU (Russian Federation), SE (Sweden), SI (Slovenia), SK (Slovakia), UA (Ukraine).

**Table 3:** 2021/22 estimated prevalence of weekly/daily alcohol consumption by country and gender.

Country	Boys	Girls
AM	20.17	10.66
AT	21.19	15.95
BE-VLG	20.00	13.91
BE-WAL	19.30	15.52
BG	42.41	16.25
CA	16.25	10.76
CH	17.21	10.62
CZ	21.21	15.83
DE	17.95	10.77
DK	20.81	15.89
EE	18.23	10.74
ES	16.34	12.82
FI	9.57	10.61
FR	17.79	10.62
GB-ENG	18.10	15.86
GB-SCT	19.52	15.89
GB-WLS	18.50	15.84
GL	9.64	10.61
GR	24.99	16.03
HR	32.24	15.83
HU	27.02	10.66
IE	9.95	10.62
IL	18.52	10.62
IS	9.43	10.60
IT	28.85	16.04
LT	15.52	10.62
LU	16.99	10.69
LV	16.45	10.62
MK	17.42	10.62
MT	26.80	15.93
NL	19.62	14.50
NO	9.67	10.62
PL	10.68	10.62
PT	12.27	10.62
RO	23.04	10.62
RU	16.99	10.62
SE	9.86	10.62
SI	26.45	16.10
SK	20.58	10.79
UA	24.15	15.96

**Table 4:** Comparison between 2017/18 estimates and observed prevalence (%) among boys.

Country	Observed	Predicted	AE	Cluster
CH	11.17	12.61	1.44	1
EE	11.34	13.56	2.22	1
IL	17.70	13.73	3.97	1
LT	9.01	10.71	1.71	1
LV	6.77	11.86	5.09	1
RU	6.03	12.52	6.49	1
AM	22.89	17.05	5.84	2
AT	23.86	18.99	4.87	2
BE-VLG	18.46	18.06	0.40	2
BE-WAL	16.17	17.57	1.40	2
CZ	18.93	18.97	0.04	2
DK	25.27	18.86	6.41	2
GB-SCT	13.22	17.59	4.37	2
GB-WLS	13.95	17.60	3.65	2
SK	20.35	17.79	2.56	2
IE	6.52	4.81	1.71	3
NO	6.85	4.40	2.45	3
SE	7.53	4.71	2.82	3
HR	29.22	27.54	1.68	4
IT	30.31	25.46	4.85	4
MT	26.13	24.08	2.05	4
SI	20.11	23.73	3.62	4
BG	49.70	34.81	14.89	5
Overall AE	Mean	Median	SD	Min–Max
	3.68	2.82	3.05	0.04–14.89

**Table 5:** Comparison between 2017/18 estimates and observed prevalence (%) among girls.

Country	Observed	Predicted	AE	Cluster
AM	5.48	5.80	0.32	1
CH	3.81	5.67	1.86	1
EE	6.31	5.88	0.43	1
IE	5.03	5.68	0.65	1
IL	6.60	5.67	0.92	1
LT	7.12	5.68	1.44	1
LV	5.43	5.68	0.25	1
NO	2.74	5.68	2.93	1
RU	2.07	5.89	3.83	1
SE	5.15	5.67	0.53	1
SK	10.39	5.99	4.40	1
AT	18.42	13.39	5.03	2
BE-VLG	7.94	10.69	2.75	2
BE-WAL	8.45	12.95	4.51	2
BG	35.09	13.83	21.26	2
CZ	11.86	13.23	1.37	2
DK	17.81	13.63	4.17	2
GB-SCT	12.32	13.62	1.30	2
GB-WLS	12.42	13.65	1.23	2
HR	14.31	13.23	1.08	2
IT	17.61	13.64	3.97	2
MT	18.70	13.69	5.01	2
SI	14.17	13.70	0.47	2
Overall AE	Mean	Median	SD	Min–Max
	3.03	1.44	4.31	0.25–21.26

## Data Availability

The HBSC International Coordinating Centre is based at the University of Glasgow, UK. Data from the HBSC study can be obtained from the HBSC Data Management Centre in accordance with the HBSC data access policy. Further information on accessing HBSC data is available from: https://www.uib.no/en/hbscdata (accessed on 30 January 2022).

## Appendix A

**Table A1:** Participation in HBSC waves from 1986 to 2018, by country. In the table, numbers represent how many 15-year-olds answered questions about alcohol consumption. Countries participating in at least 3 surveys (one of which in 2014 or 2018) were included in the analysis highlighted in green.

Region	1986	1990	1994	1998	2002	2006	2010	2014	2018
AL								1667	
AM							156	105	204
AT		974	1622	1346	1223	1476	1785	1236	1254
AZ									1274
BE-VLG			1314	1512	1967	1477	1199	1680	1366
BE-WAL	1099	795	1489	599	1349	1380	1288	1891	1596
BG						1658		1619	1453
CA		533	486	2362	1169	2245	5166	4780	
CH	1638			1789	1452	1455	2157	2156	2327
CZ			342	1172	1647	540	1513	1737	3683
DE			223	1535	1660	2492	1599	2043	
DK			482	1499	1349	1448	1189	1240	737
EE			1082	535	1267	692	853	735	1080
ES			1458		1739		1972	3645	
FI	1081	894	1132	1444	1716	1650	2077	1915	
FR			1245	1157	2538	2168	1390	1366	
GB-ENG				437	1718	1430	1059	1535	
GB-NI			1240	952					
GB-SCT	1493	1138	1326	1706	1136	2053	2500	1765	1364
GB-WLS	1975	1881	1230	1394	1144	1154	1583	1407	4117
GE									170
GL			28	409	223	383	359	21	
GR				1312	1301	1363	1615	1296	
HR					1428	1611	2406	1845	1967
HU	1185	2091	1735	732	1299	1173	1716	1084	
IE				1343	832	1582	1567	1411	1008
IL			1309	1360	1473	1721	1315	1566	2670
IS						1837	3593	3235	
IT					1211	1321	1520	1251	1278
KZ									1483
LT			1575	1257	1885	411	367	167	1134
LU						1415	1315	1030	
LV			132	1176	1083	1304	1329	1693	1313
MD								1545	
MK					1368	1880	1492	1412	
MT					627	154		185	156
NL					1239	468	1423	1319	
NO		123	133	1551	1551	840	1212	869	620
PL		96	237	1609	2110	1864	1395	1466	
PT				1186	782	1332	1423	1329	
RO						1290	1924	1405	
RS									1576
RU			1354	1322	2558	1669	1757	1388	1471
SE	133	1071	170	949	1195	1407	1960	2681	1494
SI					1046	963	991	1034	1359
SK			199	804		875	1888	1797	1192
TR						135			
UA					1585	938	1824	1628	
US				1753	1565	962	1833		

Highlight the countries that met the inclusion criteria of the study (green) and those that didn–t (red).
